# Mandibular-Derived Monocytes from 1-Year-Old Mice Have Enhanced Osteoclast Differentiation and Differentially Regulated Gene Expression Compared to Femur-Derived Monocytes

**DOI:** 10.3390/biology14030273

**Published:** 2025-03-07

**Authors:** Emilyn D. Asinas, Rachel Clark, Jadyn Nelson, Juan E. Abrahante Llorens, Kim Mansky, Amy Tasca

**Affiliations:** 1 Division of Orthodontics, Department of Developmental and Surgical Sciences, University of Minnesota School of Dentistry, Minneapolis, MN 55455, USA; 2 Oral Biology Graduate Program, University of Minnesota School of Dentistry, Minneapolis, MN 55455, USA; phil1001@umn.edu; 3 School of Dentistry Summer Research Program, University of Minnesota School of Dentistry, Minneapolis, MN 55455, USA; nel01362@umn.edu; 4 Minnesota Supercomputing Institute, University of Minnesota, Minneapolis, MN 55455, USA; abrah023@umn.edu

**Keywords:** monocytes, osteoclasts, osteogenesis, electron transport, respiratory chain

## Abstract

It is well established that both men and women lose bone as they age. While recent studies suggest unique molecular signatures of mineral-resorbing cells at different anatomical locations, most studies focus on long bones, and little is known about craniofacial osteoclasts, especially during the aging process. To determine differences between osteoclasts at different skeletal sites, we analyzed the differentiation potential, demineralization activity, and gene expression of osteoclast precursors from 1-year-old male and female C57Bl/6J mice. In our study, we determined that mandibular-derived osteoclasts were larger in size compared to those in the femur but were significantly fewer in number. However, femur-derived osteoclasts demineralized larger and more numerous areas of a calcium phosphate surface compared to mandibular-derived osteoclasts. Bulk RNA sequencing demonstrated that the mandibular-derived monocytes were enriched for genes in the WNT signaling pathway, biomineralization, and osteogenesis pathways, while femur-derived monocytes were enriched for genes in the mitochondrial respiratory complex I. Overall, our data suggest that there are different mechanisms that regulate osteoclasts from different skeletal sites as we age. This information may help to guide the design of treatments to prevent aging-induced bone loss.

## Simple Summary

Osteoclasts are the cells that resorb bone and increase their activity as men and women age. This study investigates if osteoclasts from different skeletal sites change via similar mechanisms during aging. We determined that osteoclasts from the bones of the face of older mice are larger in size but have decreased activity compared to osteoclasts from leg bones. Transcriptomic analysis revealed that different pathways are up or downregulated in facial-derived osteoclasts compared to leg-derived osteoclasts. This information is important so that we can begin to understand if there are skeletal site-specific changes that occur in osteoclasts during the aging process. Lastly, this information may eventually lead to a better understanding of the mechanisms that regulate skeletal conditions such as osteoporosis or periodontal disease, which occur more frequently as a person ages.

## 1. Introduction

The skeleton serves as the structural foundation of the body that supports mobility and bodily functions and protects vital organs. In addition to its mechanical role, it also regulates hematopoiesis, mineral homeostasis, and endocrine function. The bones of the skeleton are composed of a mineralized extracellular matrix containing osteoblasts and osteocytes [1]. Bones are further differentiated at the tissue level into cortical, a strong and compact type of bone found on the outer layers, and trabecular bone, or cancellous bone, which is found at the epiphyses and metaphysis of long bones or the internal portion of flat bones. Bones, joints, and connective tissues function as an intricate network that undergoes continuous adaptation and remodeling throughout life, reflecting the dynamic reciprocity between genetic factors, environmental influences, and physiological demands. Bone mass and shape continuously adapt to the variations in load caused by physical activity, mechanical force, hormones, nutrients, and several additional osteogenic signaling molecules [2]. Bone remodeling is critical for skeletal maintenance and requires a balance between the activity of bone resorbing osteoclasts and bone-forming osteoblasts.

Osteoclasts are multinucleated cells of hematopoietic origin that play a pivotal role in the bone remodeling process, resorbing old or damaged bone tissue to facilitate skeletal adaptation and repair. Osteoclasts result from the fusion of mononuclear precursors derived from the monocyte/macrophage lineage [3]. Both mouse and human monocytes form osteoclasts when cultured in the presence of the macrophage colony-stimulating factor (M-CSF) and receptor activator of NF-κB ligand (RANKL) released by osteoblasts and osteocytes [4]. The processes of osteoclast formation and bone resorption have been viewed as energy-intensive steps that require active metabolic reprogramming [5]. Bone resorption makes up only one side of the bone remodeling process, with osteoblasts responsible for bone deposition. It has been demonstrated by multiple studies that osteoclasts secrete or release from the bone matrix factors that regulate osteoblast differentiation and activity [6]. The precise coordination of activities between osteoclasts and osteoblasts allows for bone remodeling to be accomplished and sets up the delicate balance essential for skeletal homeostasis [7].

Aging is one of the several factors that impact the differentiation and activity of osteoclasts, which may be manifested as bone loss. It is known that both men and women exhibit bone loss with advancing age. Multiple groups report that the general trend toward bone loss persists throughout the skeleton, although some bones, such as those with a large proportion of trabecular bone, are affected earlier and more severely than others [8,9,10,11]. Bone loss with age may manifest in the oral cavity as excessive alveolar ridge resorption, chronic destructive periodontal disease, tooth loss, and referred maxillary sinus pain or fracture [8]. Kribbs et al. found a significant correlation between skeletal osteopenia and residual ridge and alveolar bone density, particularly in the edentulous mandible [12]. Different skeletal sites are also subjected to different forms of stimulation and exhibit distinct inflammatory processes in response. For example, occlusal stress stimulation and tooth-derived inflammatory responses, which exist only in the alveolar bone, affect metabolism as well as remodeling [13,14,15,16]. Therefore, the different physiological reactions, pathological responses, and clinical presentations between mandibular bone and long bones reveal the fundamental differences that exist. Many of the recent publications supporting the distinct population of osteoclasts between skeletal sites were performed in younger populations of mice with a lack of comparison to its effects in an older population. Despite the amount of literature previously mentioned, a gap in knowledge remains regarding cellular populations found at these skeletal sites and how they may change with age.

To identify differences in aging mechanisms at different skeletal sites, we performed an analysis of monocytes (i.e., osteoclast precursors) and osteoclasts from 1-year-old C57Bl/6J mice. We chose to analyze changes in 1-year-old mice as these mice are similar to 38–47-year-old humans and represent early changes that are occurring due to aging. Around this time frame, women have been shown to have declining BMD and elevated bone resorption rates [17,18]. In the current study, we determined that mandibular-derived osteoclasts from 1-year-old mice were larger in size but demineralized a calcium phosphate surface less compared to femur-derived osteoclasts. Bulk RNA sequencing of the femur- and mandibular-derived monocytes from 1-year-old mice revealed that mandibular-derived monocytes have an increase in genes involved in osteogenic pathways, while femur-derived monocytes have an increase in mitochondrial complex I and chromatin remodeling pathways. Overall, the data presented suggest that mechanistically, there are differences in osteoclast precursors at different skeletal sites as we age.

## 2. Materials and Methods

Ethical considerations. The use and care of the mice used in these study procedures were reviewed and approved by the University of Minnesota Institutional Animal Care and Use Committee, IACUC protocol number 2402-41820A. Euthanasia was performed by CO_2_ inhalation. All the mice groups were housed in a light/dark cycle (6 a.m.–8 p.m. light) in a temperature- (21.5 ± 1.5 °C) and humidity-controlled (30–70%) room and had free-to-access water and standard mouse chow.

**Figure 1:**
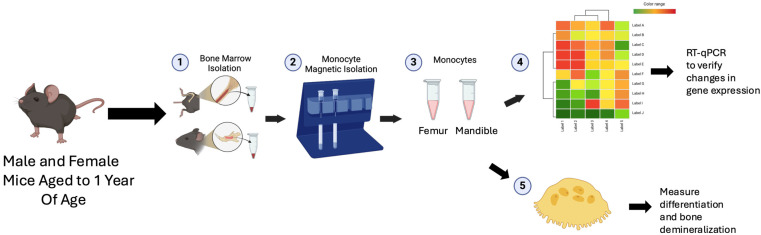
Diagram of study design. C57Bl/6 mice were aged to 1 year. (1) Bone marrow was isolated from mandible or femur. (2–3) Monocytes were isolated from bone marrow (4) Bulk RNA-Seq was performed for monocytes (5) Monocytes were cultured in M-CSF and RANKL to analyze osteoclast differentiation and demineralization ability.

Primary osteoclast cultures from bone marrow. Primary bone marrow cells were harvested from the femurs and mandibles of 1-year-old male and female C57Bl/6J mice. Bone marrow cells from the femur and mandible were cultured overnight in osteoclast media supplemented with a 1.5–2% CMG 14-12 supernatant. CMG 14-12 is a mouse cell line that expresses high levels of M-CSF. The CMG 14-12 cell line was obtained from Dr. Sunao Takeshita, Nagoya City University, Nagoya, Japan [19]. Twenty-four hours later, the non-adherent cell population, including osteoclast precursor cells, was separated from the adherent cells and re-plated in 24-well plates (MidSci) with 2 × 10^4^ cells/cm^2^ in osteoclast media supplemented with a 1.5–2% CMG 14-12 culture supernatant. Two days later, cells were re-fed with a 1.5–2% CMG 14-12 culture supernatant and 7 ng/mL of the receptor activator of the NF-κB ligand (RANKL) (R and D Systems, Minneapolis, MN, USA, catalog #462-TEC-010) to stimulate osteoclast differentiation. Cultures were fed every other day for up to 4 days for differentiation experiments (Figure 1).

Primary osteoclast cultures from monocytes. Male and female C57BL/6J mice aged up to 1-year-old were utilized. Primary bone marrow cells were harvested from the mandible and femurs of 1-year-old mice. Once the mandibles and femurs were dissected and adherent tissue was removed, the marrow was flushed from the inner compartments. Bone marrow monocyte selection was performed on total bone marrow. Monocytes were selected following the protocol of the mouse monocyte isolation kit (Miltenyi biotec, Auburn, CA, USA, catalog #130-100-629). Briefly, flushed cells were pelleted and resuspended in MACS buffer (phosphate-buffered saline (PBS) at pH 7.2, 0.5% bovine serum albumin (BSA), and 2 mM EDTA). The cells were then incubated with an FCR Blocking Reagent and Monocyte Biotin-Antibody Cocktail. After five minutes, the cells were rinsed with MACS buffer, pelleted, and resuspended in MACS buffer. The cells were incubated with Anti-Biotin Microbeads for ten minutes. After this incubation period, the cells were applied to the MACS columns in a magnetic field. Flow through was collected as isolated monocytes. The columns were rinsed three times to collect unbound monocyte cells. The monocyte cells were plated in 24-well plates (MidSci, St Louis, MO, USA) at 2 × 10^4^ cells/cm^2^ in osteoclast media supplemented with a 1.5–2% CMG 14-12 culture supernatant overnight. The next day, cells were re-fed with a 1.5–2% CMG 14-12 culture supernatant and 7 ng/mL RANKL (R and D Systems, catalog #462-TEC-010) to stimulate osteoclast differentiation. Cultures were fed every other day for up to 4 days for differentiation experiments.

Quantitation of Osteoclast Gene Expression. RNA was isolated from cells plated in duplicate using the Trizol reagent (Ambion, Life Technologies, Waltham, MA, USA) and quantified using UV spectroscopy. cDNA was prepared from 1 μg RNA using the iScript cDNA Synthesis Kit (Bio-Rad, Hercules, CA, USA) as per the manufacturer’s protocol. qPCR samples were prepared with 1 μL of the cDNA of monocyte RNA in combination with 19 microliters of a master mix containing 1 μL of the reverse and forward primers of an osteoclast marker, 8.8 microliters of water, and 10 μL of iTaq Universal Sybr Green. HPRT was used as the control gene. The PCR conditions were as follows: 95 °C for 3 min, and 40 cycles of 94 °C for 15 s, 56 °C for 30 s, and 72 °C for 30 s, followed by melting curve analysis (95 °C for 5 s, 65 °C for 5 s and then 65 °C to 95 °C with a 0.5 °C increase every 5 s). Primer sequences are shown in Table 1.

TRAP staining of osteoclast cultures. Monocytes were cultured to the desired time point and fixed in 4% paraformaldehyde for 20 min at 4 °C and then washed in PBS. Cells were stained with the TRAP buffer containing 5 mL of 0.1 M Acetate Buffer, 1 mL of 0.3 M tartrate, 100 μL of 10 mg/mL Napthol, 20 μL of Triton X-100, 3.9 mL of water, and 3 mg of Fast Red Violet. The TRAP-stained multinuclear osteoclasts were photographed with light microscopy, and the size and number of multinuclear TRAP-positive cells were quantified by an NIH ImageJ version 1.0.

Bulk RNA Seq. Monocytes were collected from 1-year-old male and female C57Bl/6J mice. Cells were lysed, and total RNA was collected using the RNA Plus Mini Kit (Qiagen, Redwood City, CA, USA, catalog #74134) following the manufacturer’s instructions. The UMN Genomics Center performed high-throughput RNA sequencing. A total of 2 × 50 bp FastQ paired-end reads for six samples (n = 62.1 million average reads per sample) were trimmed using Trimmomatic (v 0.33) enabled with the optional “-q” option: 3 bp sliding-window trimming from the 3′ end requiring minimum Q30. Quality control on raw sequence data for each sample was performed with FastQC (Version 12.0). Read mapping was performed via Hisat2 (v2.1.0) using the mouse genome (GRCm38 v94) as reference. Gene quantification was performed via Feature Counts for raw read counts. Differentially expressed genes were identified using the edgeR (negative binomial) feature in CLCGWB (Qiagen, Redwood City, CA, USA) using raw read counts as the input. The generated list was filtered based on a minimum 2× Absolute Fold Change and FDR-corrected *p* < 0.05. Data are available in Geo GSE285067. Differential pathway analysis was performed using DAVID analysis [20,21]. Heatmaps were plotted by https://www.bioinformatics.com.cn/srplot (accessed on 4 March 2025), an online platform for data analysis and visualization.

Measurement of bone demineralization. Primary bone marrow monocytes were plated on Cosmo Bio Bone Resorption surface plates (Cosmo Bio Ltd., Carlsbad, CA, USA, catalog #CSR-BRA-S96Kit) at a concentration of 3000 monocytes per well and were allowed to fully differentiate. Cells were initially plated with osteoclast media supplemented with 1.5–2% CMG 14-12 conditioned media and subsequently given osteoclast media at a pH of 6.8 supplemented with 1.5–2% CMG 14-12 conditioned media and 7 ng/mL of RANKL every two days. After 7 days of RANKL stimulation, the media was aspirated off, and 10% bleach was added to each well and allowed to sit for 10 min at room temperature. The bleach solution was removed, and each well was washed with dH_2_O twice and allowed to dry at room temperature. Plates were imaged and photographed using light microscopy. Demineralization areas were analyzed using NIH ImageJ.

Statistical analysis. All results are expressed as means with standard deviations. For all in vitro experiments, graphs represent an average of at least three independent experiments performed in triplicate. The in vivo data represent all the samples gathered and graphed together. None of the samples were removed as outliers. Student *t*-tests were used for all experiments. All statistical analyses were performed using GraphPad Prism 10 version 10.1.0.

## 3. Results

### 3.1. Mandibular-Derived Osteoclasts from 1-Year-Old Mice Are Larger than Femur-Derived Osteoclasts

**Figure 2:**
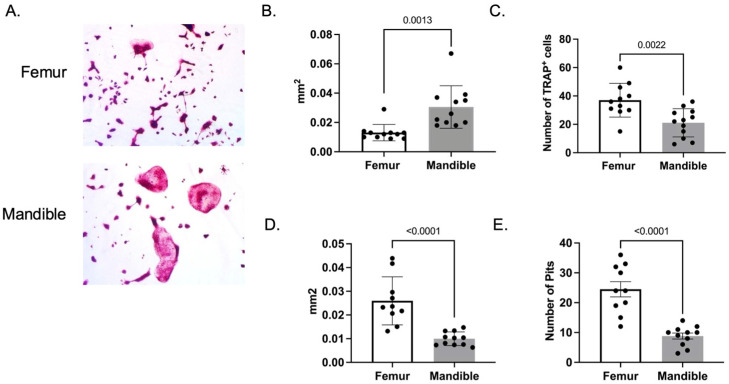
Mandibular-derived osteoclasts are larger in size compared to femur-derived osteoclasts. Monocytes were isolated from the bone marrow of the femur and mandible of 1-year-old C57Bl/6J male and female mice. Monocytes were cultured in M-CSF and RANKL to stimulate osteoclast differentiation. (**A**) Representative images of TRAP-stained osteoclasts. (**B**) The average size and (**C**) number of TRAP-positive multinuclear cells. (**D**,**E**) Monocytes were cultured on calcium phosphate-coated plates with M-CSF and RANKL for 7–10 days. (**D**) The average size of demineralized areas, and (**E**) the number of demineralized areas. The data shown are from at least three independent experiments. Samples were compared using Student’s *t*-test.

The Mansky lab has previously demonstrated that osteoclasts differentiated from the bone marrow of the mandible are larger in size compared to osteoclasts differentiated from the bone marrow of the femur [22]. However, these experiments were performed with 2-month-old mice. To determine if the same was true of mice undergoing the aging process, we isolated bone marrow from the mandible and femur of male and female 1-year-old mice, isolated monocytes, and differentiated the cells into osteoclasts in the presence of the macrophage colony-stimulating factor (M-CSF) and receptor activator of the NF-κB ligand (RANKL). Mandibular-derived osteoclasts from 1-year-old mice were significantly fewer in number (Figure 2C, *p* = 0.0022) compared to the femur but approximately twice as large (Figure 2B, *p* = 0.0013). However, when femur- and mandible-derived monocytes were plated on calcium phosphate-coated plates and treated with M-CSF and RANKL, wells containing femur-derived osteoclasts resulted in both enhanced size and a number of demineralization sites (Figure 2D,E, *p* < 0.0001). These data suggest that osteoclasts derived from the mandibular bone marrow of 1-year-old mice are larger in size but less active compared to femur-derived osteoclasts. Future experiments will need to determine if the variation in activity between femur- and mandibular-derived osteoclasts is due to differences in adhesion or substrate specificity.

### 3.2. Mandible Differentially Regulated Genes Are Conserved in Aged Mice

**Figure 3:**
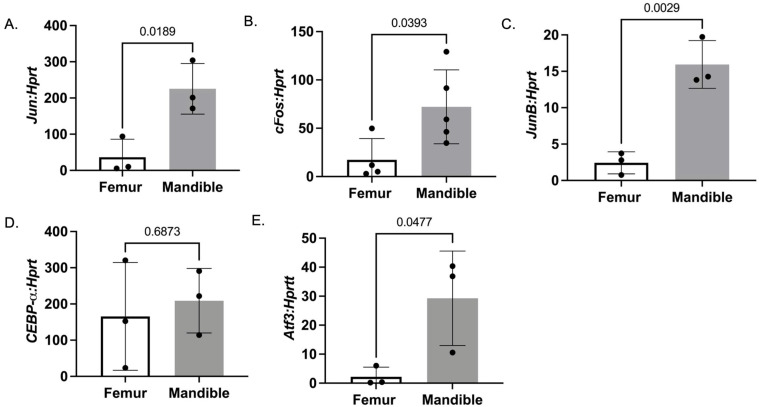
Mandibular-derived monocytes express higher levels of osteoclast transcription factors. Monocytes were isolated from the bone marrow of the mandible and femur of 1-year-old C56Bl/6J male and female mice. RNA was isolated, and qRT-PCR was performed to measure gene expression. The relative expression of (**A**) *Jun*, (**B**) *c-Fos*, (**C**) *JunB*, (**D**) *Cebp-a*, and (**E**) *Atf3.* The data shown are from three independent experiments. Data are graphed relative to *Hprt.* Samples were compared using Student’s *t*-test.

In our previous study of monocytes and osteoclasts from 2-month-old mice, we determined that the expression of lineage transcription factors *c-fos*, *junB*, and *c-jun* increased in mandibular-derived monocytes [22]. To determine if these same differentially regulated genes changed in 1-year-old animals, we isolated RNA from monocytes from the femur and mandible bone marrow and performed qRT-PCR. All three lineage transcription factors, *jun* (*p* = 0.0189), *c-fos* (*p* = 0.0393), and *junB* (*p* = 0.0029), were significantly increased in mandibular-derived monocytes compared to femur-derived monocytes (Figure 3A–C). Unlike in the monocytes from 2-month-old mice, we did not determine any significant change in *Cebp-a* (Figure 3D, *p* = 0.6873), but we did determine a significant change in *Atf3* expression (Figure 3E, *p* = 0.0477). Overall, the data support our previous observation of a difference in gene expression between osteoclasts in the craniofacial and appendicular sites. Transcription factors *c-Fos* and *Atf3* have been shown to have enhanced expression in inflammatory diseases such as arthritis, which may suggest a reason for their enhanced expression in mandibular-derived monocytes [23]. Lastly, the data demonstrated that differences in the expression of lineage transcription factors such as *c-fos* are conserved between “young” (2-month-old) and “aged” (1-year-old) mandibular-derived osteoclasts [22]. In future experiments, epigenetic changes at the promoter sites of these osteoclast genes will need to be determined, as well as if epigenetic modifications change during aging.

### 3.3. Expression Profile and Differential Pathway Analysis of RNA Collected from Mice

**Figure 4:**
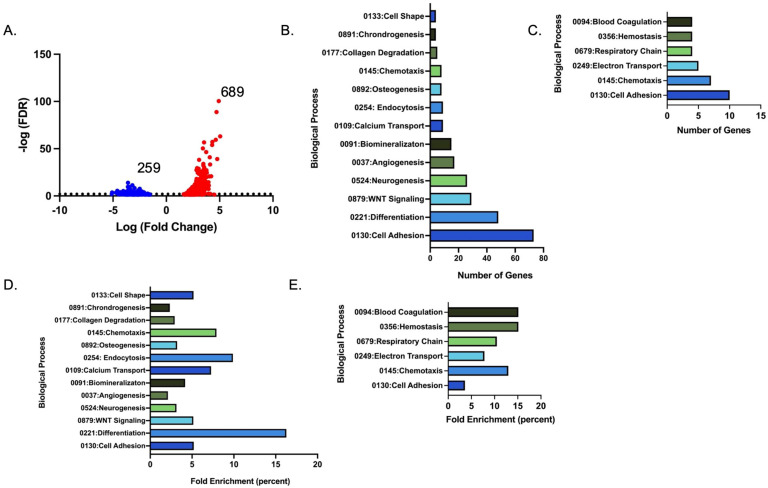
Analysis of bulk RNA-SEQ data from monocytes of 1-year-old mice. (**A**) Volcano plot showing number of differentially regulated genes identified by bulk RNA sequencing of femur- and mandibular-derived monocytes. Red dots represent upregulated and blue dots represent down regulated genes as identified by bulk RNA-SEQ. DAVID analysis of (**B**) number of genes in each upregulated pathway; (**C**) number of genes in each downregulated pathway in biological processes in mandibular-derived monocytes; (**D**) percentage of enrichment in genes in each upregulated pathway; and (**E**) percentage of enrichment in genes in each downregulated pathway in biological processes in mandibular-derived monocytes.

To obtain an unbiased understanding of the differences in mandibular- and femur-derived monocytes (i.e., osteoclast precursors), we performed the bulk RNA sequencing of monocytes isolated from the bone marrow of the mandible and femur of male and female 1-year-old C57Bl/6J mice. Using the femur expression as the baseline, we determined 948 genes to be differentially expressed. Of the differentially expressed genes, 689 genes were upregulated, and 259 genes were downregulated in the mandible monocytes compared to the femur (Figure 4A). Using DAVID analysis, the most enriched pathways upregulated in the mandible monocyte population included WNT signaling, biomineralization, and cell-adhesion pathways (Figure 4B,D). Signaling pathways differentially regulated in the mandible monocytes include WNT, BMP, calcium, Hippo, Hedgehog, TGF-β, and PI3K. WNT and TGF-β signaling in monocytes has been shown to activate signals that serve as a chemoattractant, such as CCL2 [24,25]. The upregulation of these pathways in the mandibular-derived monocyte population suggests that there is an expression of genes that promote monocyte migration and attraction to inflammatory sites. When determining the downregulated pathways in the mandibular-derived monocytes, metabolism (mitochondria complex I genes), cell adhesion, ATP-dependent chromatin remodeling, and chemotaxis appeared to be the most changed pathways (Figure 4C,E). Genes expressed in the mitochondria complex I were shown to suppress inflammation and promote osteoclast differentiation [26].

### 3.4. Mandible-Derived Monoctyes and Osteoclast Precursors Express Increased Osteogenic Factors

**Figure 5:**
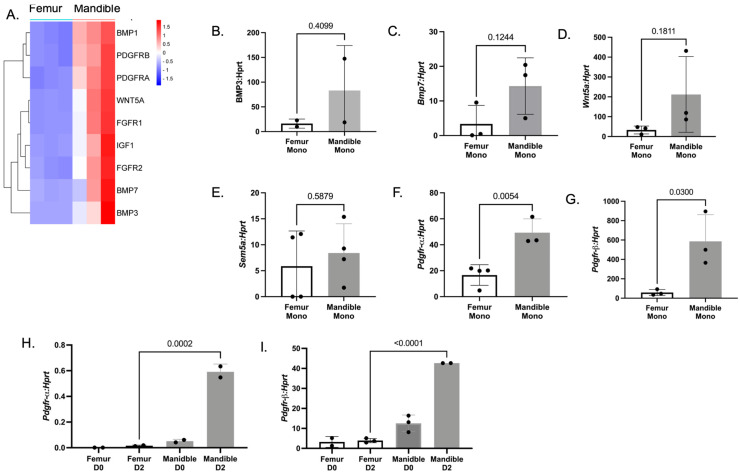
Osteogenic molecules are enhanced in expression from mandibular-derived monocytes. (**A**) Heat map of osteogenic genes identified in bulk RNA-SEQ of 1-year-old mandibular-derived monocytes. (**B**–**G**) qRT-PCR of selected osteogenic genes identified by RNA-SEQ of mandibular-derived monocytes. Individual *p*-values are as shown. (**H**,**I**) qRT-PCR of selected osteogenic genes from mandibular- and femur-derived osteoclast precursors treated with either M-CSF only (Day 0) or M-CSF and RANKL (Day 2). Data shown are from three independent experiments. Data are graphed relative to *Hprt.* Samples were compared using Student’s *t*-test.

Monocytes were isolated from the mandible and femur of 1-year-old mice. Differentially regulated genes involved in biomineralization were analyzed by qRT-PCR. There was a trend for an increase in *Bmp3* (Figure 5A, *p* = 0.4099), *Bmp7* (Figure 5B, *p* = 0.1244), *Wnt 5a* (Figure 5C, *p* = 0.1811), and *Sem5a* (Figure 5D, *p* = 0.5879) expressions in the mandibular-derived monocytes compared to the femur-derived monocytes; however, none of the differences were significant. Both expressions of *Pdgfr-a* (Figure 5F, *p* = 0.054) and *Pdgfr-b* (Figure 5G, *p* = 0.0300) were significantly increased in mandibular-derived monocytes compared to femur-derived monocytes. When we determined the expression of these factors on day 0 (M-CSF only) and day 2 (48 h with M-CSF and RANKL), the osteoclast precursors *Pdgfr-a* (Figure 5H, *p* = 0.002) and *Pdgfr-b* (Figure 5I, *p* < 0.001) were increased in mandibular-derived osteoclast precursors (day 2) compared to femur-derived osteoclast precursors. Both PDGFRA and PDGFRB were detected in cortical bone in a spatial study of bone-remodeling sites in humans [27]. Overall, these data suggest that mandibular-derived monocytes and preosteoclasts from 1-year-old mice have increased the expression of receptors for osteoclast coupling factors compared to femur-derived cells. Future experiments will test if “young” and “aged” mandibular-derived osteoclast precursors and/or osteoclasts are enhanced in promoting osteoblast proliferation, migration, and mineralization.

### 3.5. Femur-Derived Monocytes and Osteoclast Precursors Express Increased Electron Transport and Respiratory Chain Genes

**Figure 6:**
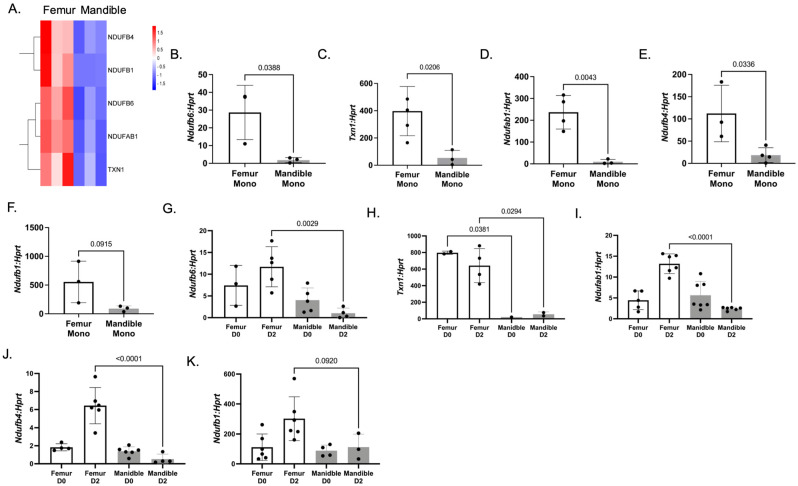
Mitochondria complex I genes are upregulated in 1-year-old femur-derived monocytes. (**A**) Heat map of mitochondria complex I genes identified in bulk RNA-SEQ of 1-year-old mandibular- and femur-derived monocytes. (**B**–**F**) qRT-PCR of selected mitochondria complex I genes identified by RNA-SEQ. Individual *p*-values are as shown. (**G**–**K**) qRT-PCR of selected osteogenic genes from mandibular- and femur-derived osteoclast precursors treated with either M-CSF only (Day 0) or M-CSF and RANKL (Day 2). Data shown are from three independent experiments. Data are graphed relative to *Hprt.* Samples were compared using Student’s *t*-test.

Femur-derived monocytes had significantly upregulated expression of *Ndufb6* (Figure 6A, *p* = 0.0388), *Txn1* (Figure 6B, *p* = 0.0206), *Ndufab1* (Figure 6C, *p* = 0.0043), and *Ndufb4* (Figure 6D, *p* = 0.0336) in femur-derived monocytes compared to mandibular-derived monocytes. These genes are involved either in cellular hypoxia and/or in complex I of the mitochondria respiratory chain. These same groups of genes were also significantly upregulated in osteoclast precursors that had been treated with M-CSF and RANKL for 2 days (compare Femur D2 to Mandible D2 in Figure 6G–K). These data suggest that these gene changes are conserved both in femur-derived monocytes and osteoclast precursors. The expression of *Ndufb1* (Figure 6E,K) in femur-derived monocytes and day 2 osteoclasts was increased but not significantly compared to mandibular-derived cells. These results propose questions such as do the expression of these genes continue to increase with aging? Are these genes necessary for the increased bone resorption seen in aging individuals? Why are these genes not increased in mandibular-derived osteoclast precursors and preosteoclasts?

## 4. Discussion

In a previous study performed by Clark et al., the osteoclast population in the mandible was found to be distinct in comparison to osteoclasts located in the femur of 2-month-old mice [22]. These general trends were also observed in the 1-year-old mice population in this study. When the mandible-derived monocytes of this mice population were differentiated into osteoclasts, the multinuclear cells were larger in size but reduced in number compared to femur-derived monocytes, similar to what was seen in 2-month-old mice by Clark [22]. However, when we analyzed the ability of the femur- and mandibular-derived osteoclasts from the 1-year-old mice to demineralize a calcium phosphate surface, we determined that femur-derived osteoclasts resulted in more and larger demineralized areas. Differential expressions of adhesion molecules may contribute to differences in demineralization capacity. Based on our bulk RNA-sequencing data, *integrin beta 5* (*Itgb5*) expression is enhanced (3.5-fold) in mandibular-derived monocytes compared to *integrin beta 3* (*Itgb3*), which is enhanced (3.3-fold) in femur-derived monocytes. *Integrin beta 3* has been shown to be necessary for bone resorption by osteoclasts [28]. Osteoclast precursors have also been shown to express *integrin beta 5,* which recognizes the same amino acid motif as *integrin beta 3*. However, the loss of *integrin beta 5* expression in an ovariectomized mice model led to enhanced *integrin beta 3* expression and the resorptive activity of osteoclasts, suggesting that the expression of *integrin beta 5* may have a negative effect on *integrin beta 3* expression and activity [29]. The difference in the ability of femur- and mandibular-derived osteoclasts to demineralize a calcium phosphate surface may be due to differences in the ability of the osteoclasts when adhering to a surface and allowing for demineralization to occur. Osteoclasts require adhesion for their survival, and this characteristic may contribute to the limited localization of osteoclasts on proper substrates such as bone surfaces in the body [30]. Lastly, a difference in substrate (bone vs. dentin) may also explain the differences in osteoclasts from the different skeletal sites to demineralize the calcium phosphate surface.

The genes that were upregulated in 1-year-old mice are associated with transcription factors involved in the same processes as those upregulated in 2-month-old mice, suggesting that genes involved in osteoclast lineage commitment are unaffected by the aging process in the mandible. These data suggest a more active state of osteoclast differentiation in the mandible, driven by lineage commitment factors. ATF3 is a transcription factor that has been shown to be necessary for osteoclast differentiation by controlling the proliferation of osteoclast precursors [31]. In monocytes, *Atf3* is responsive to IL-1β signaling and has been shown to promote monocyte/macrophage differentiation [32].

Bulk RNA sequencing data from monocytes isolated from the bone marrow of 1-year-old mice revealed differentially regulated genes in the mandible and femur. The information on transcriptome complexity unveiled that WNT signaling, biomineralization, and cell adhesion pathways are highly enriched in the mandible, while electron transport, respiratory chain, cell adhesion, and chemotaxis are downregulated in the mandibular-derived monocytes. To our knowledge, this is the first study to identify differences in gene expression in monocytes and osteoclast precursors from mice that are 1 year of age. The RANKL stimulation of osteoclast precursors for 48 h leads to an increase in mitochondrial basal respiration, ATP-linked respiration, and electron chain activity [33]. Additionally, the RANKL treatment of osteoclast precursors leads to an increase in mitochondrial content and biomass [34,35,36]. In a study performed by Taubmann et al., they noted a simultaneous increase in both glycolytic activity and mitochondrial respiration when comparing activated osteoclasts to resting osteoclasts, suggesting that bone resorption considerably increases osteoclast metabolic and energy requirements [37]. This adaptation by osteoclasts is thought to be linked to an increased demand for energy, which may also be supported by the increased expression of genes involved in complex I of the mitochondrial activity in the femur-derived monocytes and D2 osteoclast precursors of 1-year-old mice. Interestingly, estrogen also negatively regulates the expression of mitochondria complex I genes; future experiments will be performed to determine if the expression of this set of genes changes in mandibular-derived monocytes and osteoclast precursors from ovariectomized mice [33]. Mice conditionally deleted for *Ndufb4,* one of the genes that were upregulated in our 1-year-old femur-derived monocytes and preosteoclasts, had osteoclast precursors that failed to differentiate because of a shift in the Ly6C^high^, CD11b cell population, resulting in the formation of inflammatory macrophages and not osteoclasts [26].

Understanding the changes in cellular processes with aging in mice contributes to our understanding of the molecular changes at these skeletal sites in humans. Moreover, 1-year-old mice were used in this study to correlate to humans 38–47 years of age, but perhaps more significant findings would be seen in studies with mice at 18–24 months, which are comparable to humans at 56–69 years of age. Since this study removed any pathological factors in mice to solely study the effect of aging, the effect of estrogen changes on the osteoclast activity at these skeletal sites, as well as conditions of inflammation such as periodontal disease, will be considered in future experiments during the examination of the mechanisms by which aging affects osteoclast differentiation at different skeletal sites. Periodontal disease is more common in older individuals, and future experiments will need to determine the mechanisms by which mandibular-derived osteoclast precursors and osteoclasts change in the presence of enhanced inflammation and aging in the oral cavity. Identifying epigenetic changes that result in changes in gene expression during aging and inflammatory conditions may allow for better treatment options. Lastly, drugs targeting epigenetic regulators may allow for the development of therapies that are reversible and allow for the treatment of bone loss due to inflammatory conditions.

From previous research, it is unclear as to what changes we expected to see in these experiments. Two schools of thought exist to say that the craniofacial region and appendicular skeleton are similar and that these skeletal sites differ during the aging process. Many earlier research publications claim no differences between the craniofacial region and appendicular skeleton. In studying the variation in mineral density of the mandible and radius in both sexes, Henrikson et al. claimed that the changes seen in these skeletal sites with age were qualitatively the same [38]. Hildebolt et al. explained that cross-sectional QCT data of selective measures of vertebral cortical and trabecular bone demonstrated a similar rate of cortical demineralization between the skeletal sites [39]. These studies were performed on individuals with various diseases, such as osteoporosis or nutritional deficiencies, and lacked descriptions of the cellular processes within these skeletal regions. More recent research publications have shifted views with more diverse experiments to support the idea that these sites represent different cell populations. These recent studies, supporting the claim that the mandible and femur represent distinct entities, were performed on younger mice (8 weeks or 1-month-old mice). There is limited information on what happens to the cellular composition and activity at these skeletal sites at an individual age, but our study has begun to fill this gap in existing knowledge.

## 5. Conclusions

This study reveals that osteoclast precursor populations at the mandibular and femoral sites have unique changes in gene expression. The results support our hypothesis, showing differences in gene expression and increased differentiation capabilities of osteoclast precursors derived from the bone marrow of the mandible compared to the femur. The larger size and decreased number of osteoclasts found in the mandible when compared to the femur were seen in both 2-month-old and 1-year-old mice. Through bulk RNA sequencing, it was shown that different pathways affect these two skeletal sites in an aging population. This may suggest that there are different pathways regulating the craniofacial and appendicular regions during aging. The mandible and femur of 1-year-old mice exhibit distinct characteristics from each other. Through an appreciation of these differences, a better understanding of what osteoclastic changes occur with aging may be noted and utilized within treatment planning for older patients seeking medical or dental care.

## Figures and Tables

**Table 1:** Primer sequences.

Gene	Forward Sequence (5′-3′)	Reverse Sequence (5′-3′)
*Junb*	TCA CGA CGA CTC TTA CGC AG	CCT TGA GAC CCC GAT AGG GA
*cJun*	TCC CCT ATC GAC ATG GAG TC	TGA GTT GGC ACC CAC TGT TA
*cFos*	CCA AGC GGA GAC AGA TCA ACT T	TCC AGT TTT TCC TTC TCT TTC AGC AGA
*Atf3*	GAG GAT TTT GCT AAC CTG ACA CC	TTG ACG GTA ACT GAC TCC AGC
*BMP7*	TAC GTC AGC TTC CGA GAC CT	GGT GGC GTT CAT GTA GGA GT
*Ndufb1*	CTT CTA CGT GAG CAC TGG GTT	CTT CAT TGG GCC TCA GTT CCC
*Ndufb4*	GGG CAG CCC TCA TAT CCG C	GGC GCA GGC TTA TAC TTG GA
*Ndufb6*	CGG GAG CTA AGG AGA CGA TG	GCG GTA CGC CTT AAA GAC CA
*Ndufab1*	ACA CAC TGA CAA CCA AGA GTG A	TTG CGC CAA TTC TTC AGC TAC
*PDGFRA*	GCA GTT GCC TTA CGA CTC CAG A	GGT TTG AGC ATC TTC ACA GCC AC
*PDGFRB*	GAA CGA CCA TGG CGA TGA GA	GCA TCG GAT AAG CCT CGA ACA
*Sem5a*	GCC ACT TCC ATC AAA CAC GCA G	GTC ATG CTC AGA CTC TCC TCC A
*Txn1*	GCT TGT CGT GGT GGA CTT CT	GGC AGT CAT CCA CAT CCA CT
*Wnt5a*	CAA CTG GCA GGA CTT TCT CAA	CAT CTC CGA TGC CGG AAC T
*BMP3*	TAA CAC GGT CCG CAG CTT CAG A	TGT GGC TGA CAA AAT GTT CTC CG

## Data Availability

Primary data presented in this manuscript are available upon request. Bulk RNA SEQ data are available at NCBI Geo GSE285067.
